# Metropolitan and National Nursing Association

**Published:** 1894-05-05

**Authors:** 


### HOSPITAL FESTIVALS, MEETINGS, ETC.

### METROPOLITAN AND NATIONAL NURSING ASSOCIATION.

The eighteenth annual meeting of the Metropolitan and National Nursing Association for providing trained nurses for the sick poor (in connection with the Queen Victoria's Jubilee Institute for Nurses) was held on Saturday, by the kind permission of the Duke of Westminster, who is the chairman of the Executive Committee, at Grosvenor House. The Duke was, however, unfortunately called away into the country on important business, aud therefore unable to attend; the vice-chairman, Mr. W. S. Caine, M.P., was absent from a like cause, and the chair was accordingly occupied by Sir E. A. H. Lechmere, M.P. There was a large attendance, which included many ladies.

The Chairman briefly moved the adoption of the report, which had been circulated among those present. This document stated that the work continued to be carried on with much energy and vigour, and the sound principles and high standard of district nursing which had been amongst the distinguishing features of the Association from its commencement were still maintained. After giving details of the work, it set forth that on January 31st, 1893, there were nurse probationers in training, 7; ditto admitted during the year, 10; candidates, 5; total 31. Completed their training, 12; candidates sent for hospital training, 4; left as unsuitable, 3; remaining at the end of the year, 12; total, 31. All of these were trained at the expense of the Jubilee Institute, and sent as Queen's Nurses to nursing associations affiliated to the Institute in various parts of the country. The demand for trained district nurses had greatly increased of late years, stimulated by the work of that and similar associations, and by the growing recognition of the value of trained nursing in time of sickness, but the supply of suitable candidates for training in district work did not keep pace with the demand. The committee, however, kept steadily before them the principle which they had always maintained, that the sick poor should have the best nursing that could be obtained. The number of cases nursed during the year again showed a considerable increase on former years, being 1,389, as against 968 in 1892 and 761 in 1891. Besides these regular cases of home nursing there were 361 cases of children attending Board schools in the neighbourhood and suffering from sore eyes, burns, scalds, &c., which, if not properly attended to, would often develop into serious ailments. In consequence of the death of many old subscribers, it was necessary to appeal for new subscribers to fill up the vacant places of those who were gone, in order that the work, which had grown considerably as it had become more known and valued, might not be crippled for want of funds. It was now generally acknowledged to be a real boon to the working classes, and one of the soundest and best methods of helping them in time of sickness and suffering. The statement of receipts and payments for the year showed that the receipts amounted to £1,740 13s., of which sum subscriptions represented £890 19s. 6d., and that the total ordinary expenditure was £1,532 17s. 2d., though other payments left eventually only the sum of £7 16s. 1d. in hand, as against £82 18s. 9d. twelve months previously.

The Chairman, in regretting the absence of the Duke of Westminster, said they must all feel grateful to him for the cordial welcome he had that day extended to the members of the Association. He (the chairman) took part in the original inception of the Association, and he had always felt a great interest in it, for it was a type, it appeared to him more than any others, of what was the very best basis for nursing the sick poor. He thought the society had done more than any other to bridge over the gulf which separated the poor and the rich, the homes of luxury, and the homes of squalor and misery, for the nurses had been to the poor almost, he might say, a sort of angels of light, not only tending them in their sufferings, but teaching them to make their homes more clean and more comfortable to the promotion of comfort and health. He hoped the Association, as it had been already the means of spreading nursing work in this country, as it had been already the precursor of other valuable institutions, would continue for many years to come the useful work which it had done in the past. (Cheers.)

Mr. Arnold White, in seconding the motion, said that in the present day they heard very much about the inhumanity of man to man, and very much about the rights and very much about the wrongs, but very little about the duties of man. They were promised unlimited experiments in social reform, the happiness, comfort, and welfare of the masses; but there were some things which politicians could not give them—politicians could not give them that individual self-sacrifice and readiness to lay down personal comfort for the sake of others which was presented by the society. Politicians could not give them that only means of bridging the chasm between the classes and the masses, for it could only be supplied in the spirit of Christianity and religion, such as was in the hands of ladies of gentle birth. He knew of no weapon in the armoury of social reform which was so effective as the spirit of self-sacrifice, when it was carried into the squalid and miserable homes of the poor. When the poor man came to be laid aside with illness he remembered that it was not the new democracy nor the dignity of labour or any such movement which could materially help him. His troubles could only be remedied by the spirit of Christian sacrifice in the hands of ladies of gentle birth. The programme of those ladies was contained in no political programme. It must be remembered that they worked for none of the stimulus of gain, for none of the notoriety that formed so large a part of the prose of men of the world. The public did not know their names, but they did know their work, and they honoured, loved, reverenced, and admired it. The misery of the multitude was too often made the machinery of imposing politicians and social reformers, but it was not so with those ladies whose names were not known and whose work was anonymous, yet they were truly working and striving, like Horatius of old, to "keep the bridge" for the sake of England and for the sake of humanity. He could not help thinking, when he heard so much of the "revolting daughters" and their claims to individuality, whether it was possible to attain a higher ideal than was presented by those ladies whom they were there to support. A great deal of their work was thankless and ungrateful, but those present could at all events express their approval of the way in which they worked. (Cheers.)

The motion was then carried.

Mr. RATHBONE, M.P., moved: "That this meeting, recognising the good services done by the Metropolitan Nursing Association, pledges itself to support the Association in carrying on its work." He said that the nursing of the poor in their homes had caused a demand for nurses which the Association were quite unable to supply. It was sad to be obliged to say it, and it was a thing involving great responsibilities, and in no way aiding in the work; but some people, if they could not get a good nurse took a bad one, and if they took one who was incapable then the work languished, was desponding, and did not pay, whereas every good nurse sent out from the best institutions in the country was an apostle of nursing, a missioner of nursing. (Cheers.) The spirit of nursing spread with the most extraordinary rapidity all round, and created new associations, and that was why a great responsibility rested on all those who were connected with an association like the Metropolitan. He had never known an instance in the case of the associations now being founded throughout the country in which the people having put their hands to the work did not do well, where it was not continued, or where there was any disposition to go back from the work undertaken. Therefore any support they gave now was of infinitely more practical advantage than what they might do three, four, or five years' hence. The work was spreading its work in branches all over the land, which would continue to do well if those present took care to remember that on the Central Institution primarily rested the duty of keeping up the standard of work, and setting a standard of work throughout the country. (Cheers.)

Mrs. SCHARLIEB, M.D., in seconding the motion, said that if they all knew what illness meant in a well-ordered home with their admirable nursing and the necessaries and comforts that could make sickness endurable, they should think for a few moments of what illness meant in the homes of the poor, when the breadwinner was laid aside, or what was most dreadful, the mother was taken ill, and of the anxiety the parents suffered when their little ones were laid aside, and they saw them suffering pain, discomfort, and fever, knowing that they could not procure for them any alleviation, which came to the rich so readily. That was the sort of work—to relieve that anxiety and to mitigate that suffering—that the Metropolitan and National Nursing Association proposed to do, and it was a work that it had been doing for many years. The nurses were constantly helping people to maintain that wholesome feeling of self-respect which was the backbone of our English character and of our English virtues. Those nurses were educated gentlewomen, and one advantage which people of education had over others was that that general culture and imagination were more trained, and they could more easily put themselves into the place of those whom they had to do with, and they were so much more qualified to judge about the troubles in the homes of the poor which were likely to affect their patients; their general knowledge of other sorts was better; they had more knowledge not only of the treatment of illness itself, which was a very small part of the general conduct of a matter, but they were more likely to understand the management of drains and sanitation, the reasonableness of the antiseptic system, and all such things, and that was another reason in favour of the work of the Association. Another point worthy of remembrance was the absolute necessity of giving nurses the best training it was possible to give. In the days of old one could remember how very little chance the orders of the doctor had of being carried out, and how very few efforts were made to alleviate suffering, but for many years they had been trying to train nurses thoroughly. There were nurses who worked in the hospitals, nurses who worked in the private homes of the well-to-do, and nurses who worked in the homes of the poor. It was quite evident that different qualities would go to make up the ideal nurse of any one of those three orders, but personally she (the speaker) must say that if she had to be a nurse to do the easiest work that a nurse was capable of, she should choose the well-ordered hospitals, where one would have the moral support of the house surgeon and everything ready to hand—only to ask to receive. They should think what it meant to be a district nurse, who probably in her visits would often find no clean water, and in few instances boiling water. She had to improvise surgical apparatus, such as splints, and there was exceedingly little wealth, though a very great deal of wit and a very great deal of *savoir faire* behind the district nurse. Therefore such nurses needed to know a great deal, and to have the capacity of using that knowledge very much more than the nurses in the hospitals. They all agreed that the trainiug of the district nurses must be the best as far as was possible to the resources of the Association; but it was not enough to say those things, they must be prepared to do them. They must be ready, as far as in them lay, to help the Association and all other associations to alleviate the sorrows and sicknesses of the poor. (Cheers.)

Mr. BURDETT and the Rev. W. H. CALDWELL supported the resolution, which was carried unanimously.

Mr. F. D. MOCATTA proposed a resolution electing as members of the Association for 1894-5 certain ladies and gentlemen whose names figured in the report, and in so doing congratulated those present on the success which the Institution had attained. It was not only a great work in itself, but it had been the beginning of other kindred great works. (Cheers.)

Mr. H. BONHAM-CARTER, in seconding the resolution, sketched the origin of the Association, saying that the Jubilee Fund had given a tremendous impetus to the nursing movement throughout the country, so that there was a considerable danger that the standard of nursing would diminish to some extent from the impossibility of obtaining a sufficient number of thoroughly-trained nurses. The object of the Association was to still maintain its high standard of training, and to extend its work, but it wanted money, and he thought that it would be money spent in the very best possible way. He thought that the nurses turned out of those homes were far superior to all others, and with reference to the question of surgical training, he might mention that they were desirous of increasing the minimum training period of a year, but unless the subscription list was enlarged they should not be able to extend their sphere of usefulness.

The motion was then carried, and the meeting concluded with a cordial vote of thanks to the Duke of Westminster, proposed by the Rev. DACRE CRAVEN, for permission to hold the gathering in Grosvenor House.

